# Acquired Gerbode Defect Diagnosed Seven Years After Surgical Mitral Valve Repair for Severe Primary Mitral Valve Regurgitation: A Case Report

**DOI:** 10.7759/cureus.94436

**Published:** 2025-10-13

**Authors:** Hasan Kazma, Suzan Iskandar, Joe Hitti, Abbas Rachid, Malek Mohammed

**Affiliations:** 1 Cardiology, Bahman University Hospital, Beirut, LBN; 2 Cardiology, Lebanese University Faculty of Medicine, Beirut, LBN; 3 Internal Medicine, Lebanese University, Beirut, LBN; 4 Cardiovascular Disease, Bahman University Hospital, Beirut, LBN

**Keywords:** complications of surgical mitral valve repair, echocardiography and color doppler, gerbode defect, mitral valve prolapse, mitral valve replacement, mri cardiac, myxomatous mitral valve disease, percutaneous repair of primary mitral regurgitation, primary mitral regurgitation, surgical mitral valve repair

## Abstract

Surgical mitral valve repair is the main treatment for patients with severe primary mitral regurgitation who are symptomatic; this technique is usually done in patients with myxomatous degeneration of the mitral leaflets, such as mitral valve prolapse. In these patients, surgical mitral valve repair is recommended over surgical mitral valve replacement; also, surgical mitral valve repair is recommended over percutaneous repair in patients with acceptable surgical risk. We report herein the case of a 54-year-old man who underwent mitral valve repair seven years previously for myxomatous anterior mitral valve prolapse and severe regurgitation. The patient was asymptomatic at the current follow-up; however, his color Doppler echocardiogram showed grade II mitral regurgitation with a shunt between the left ventricular outflow tract and the right atrium, just above the tricuspid septal leaflet, known as an acquired Gerbode defect, a rare entity.

## Introduction

Patients suffering from chronic mitral regurgitation are divided into two groups: a first group with "primary mitral valve disease," as an example, myxomatous mitral valve prolapse or rheumatic mitral valve disease; and a second group with "secondary or functional mitral valve disease," as an example, ischemic mitral regurgitation or mitral regurgitation secondary to dilated cardiomyopathy [1]. Severe primary mitral regurgitation is mainly defined by the following echocardiographic and color Doppler criteria: vena contracta (VC) > 0.7 cm, regurgitant volume > 60 ml, effective regurgitant orifice > 0.4 cm², regurgitant fraction > 50% [1].

In patients with chronic mitral regurgitation due to primary mitral disease with myxomatous valve degeneration and prolapse, surgical mitral valve repair/replacement or percutaneous repair is indicated in (1) symptomatic patients with severe mitral regurgitation (in these cases, surgical mitral valve repair is recommended over replacement); (2) asymptomatic patients with severe mitral regurgitation and left ventricular ejection fraction (LVEF) <60% or left ventricular end systolic diameter (LVESD) > 4.0 cm (in these cases, surgical mitral valve repair is recommended over replacement); (3) asymptomatic patients with severe mitral regurgitation and normal LVEF or with LVESD < 4 cm (in these cases, surgical mitral valve repair is the only recommended procedure); (4) symptomatic patients when the left ventricle (LV) shows negative remodeling over three measurements like a decrease in LVEF and an increase in LV size (in these cases, both surgical mitral valve repair and replacement may be performed); and (5) asymptomatic patients with preserved LV function but who develop atrial fibrillation or resting pulmonary hypertension (in these cases, surgical mitral valve repair is recommended over replacement [1]. When surgical mitral valve repair can be performed with near-zero percent residual regurgitation and less than 1% mortality, it is then recommended over replacement [1].

In symptomatic patients with severe primary mitral regurgitation and high surgical risk, percutaneous transcatheter edge-to-edge repair is recommended over surgery if the patient's life expectancy is more than one year, provided the procedure is technically feasible [1]. When less than half of the posterior leaflet is involved in the myxomatous disease process, the only recommendation is surgical mitral valve repair, provided that the patient's surgical risk is low [1]. These recommendations are due to the fact that repair of the mitral valve in patients with severe primary regurgitation has a very high success rate and good long-term outcome, especially the repair of the isolated posterior leaflet. Surgical mitral valve repair obviates the need for oral anticoagulation used with mechanical heart valve replacement, is more durable than bioprosthetic valve replacement, and has fewer operative complications compared to replacements [2-4].

The repair of the anterior myxomatous degenerative mitral leaflet in mitral valve prolapse, causing severe mitral regurgitation, mainly consists of resection of a part of the anterior mitral leaflet and annuloplasty with implantation of a flexible ring prosthesis (i.e., Carpentier-Edwards ring) and possible re-implantation of chordae tendineae [5,6]. A study by Khairallah et al. found that, in patients with severe mitral regurgitation secondary to myxomatous degeneration of the anterior mitral leaflet, surgical repair could be performed in only 55.6% of patients; this was mainly due to difficult technical repair and intraoperative decisions to replace the valve. This study also showed that 81.7% of mitral valve replacements were done without even a repair attempt. This tendency toward mitral valve replacement is more frequent in patients with myxomatous disease affecting mainly the anterior leaflet, due to technical difficulties and a fear of early recurrence of mitral regurgitation with the surgical repair technique in this patient population [6].

The presence of even mild mitral regurgitation, or the early recurrence of mild mitral regurgitation immediately after surgical mitral valve repair in mitral valve prolapse with myxomatous leaflet disease, indicates that the disease will progress, leading to severe mitral regurgitation requiring valve replacement later, when symptoms supervene. Therefore, an important predictor of mitral valve reoperation is the presence of even mild residual regurgitation after the repair procedure. This is due to the progression of the lesions with time [5-7]. The rate of recurrence of moderate mitral regurgitation (grade > 2/4) is approximately 3.6 per year, according to Flameng et al. [7]. In one cohort from Badhwar et al., the complications of surgical mitral valve repair and replacement were operative mortality 1%, operative morbidity and mortality 8.1%, stroke 1.2%, acute kidney injury 1.1%, cardiac reoperation 2.9%, prolonged ventilator >24 hours 4.6%, deep sternal infection 0.1%, postoperative atrial fibrillation 25.8%, and permanent pacemaker 3.7% [8]. The complication rate was higher among patients undergoing mitral valve replacement. In cases of conversion from repair to replacement, the complication rate was highest; the rates of repair success and complications were related to the center's and the operator's experience with this type of surgery [8].

One rare complication of surgical mitral valve repair or replacement is the acquired Gerbode defect. A Gerbode defect is an abnormal shunt between the LV and right atrium (RA). It can be congenital or acquired, like post-cardiac surgery [9-11]. It is an abnormality of development or an injury to the atrioventricular septum that leads to shunting between the LV and RA; the most common etiology is congenital. The incidence of a shunt between the LV and RA is 0.08% among all congenital defects undergoing catheterization and 0.12% in autopsy reports [11]. The congenital form is considered a high ventricular septal defect (VSD) (membranous atrioventricular septum) [9]. More cases of acquired Gerbode defect are being diagnosed due to the improvement in imaging, such as two-dimensional (2D) and 4D transesophageal echocardiography (TEE), cardiac computed tomography (CT), and cardiac magnetic resonance imaging (MRI) [11].

The incidence of postoperative shunts between the LV and RA after valve surgery is around 0.65% [11]. The tricuspid valve is located approximately 15 mm below the mitral valve. The part of the membranous atrioventricular septum between both valves is thin and may be ruptured in case of injury, thus creating a direct shunt from LV into RA. This rare acquired form of atrioventricular septal defect may result from complications of cardiac surgery, such as valve replacements (aortic and mitral), surgical closure of the VSD, endocarditis, chest trauma, myocardial infarction, and endomyocardial biopsy. During valve surgery and in cases of excessive valve annulus calcification, the surgeon has to perform major debridement, thus increasing the risk of acquiring a Gerbode defect. Redo-cardiac surgeries are associated with a higher risk of developing this defect due to adhesions. Valve endocarditis may lead to annular abscess formation, which may weaken the membranous atrioventricular septum and result in an LV-RA shunt [11].

In large LV-RA shunts, symptoms of heart failure will develop, requiring closure of the shunt. The preferred technique is percutaneous closure because surgical closure carries an increased risk (adhesions from previous surgery make the surgical closure technically difficult and increase the surgical risk) [11]. According to Riemenschneider and Moss [10], there are two types of congenital Gerbode defect: the direct type, which is a direct communication between the LV and RA through the membranous septum just above the septal tricuspid leaflet, and the indirect type, which is a VSD usually associated with tricuspid regurgitation through a perforation in the septal tricuspid leaflet [9,11]. Another terminology also exists [9]: the supra-valvar defect that occurs in the atrioventricular septum and is a direct connection between the LV and RA just above the tricuspid septal leaflet and is called type 1 [11,12]. A second type occurs between the ventricles and is known as an infra-valvar defect; the communication with the RA is through a perforated septal tricuspid leaflet and is called type 2 [11]. There is a third type with both supravalvar and infravalvar shunts. The incidences of these three types are 76%, 16%, and 8%, respectively [11].

## Case presentation

This case involves a 53-year-old patient with a history of hypertension, no diabetes mellitus, and no dyslipidemia; he is not a smoker. He underwent surgical mitral valve repair seven years ago for symptomatic primary severe mitral regurgitation due to prolapse of a myxomatous anterior mitral leaflet (A2). At that time, his preoperative coronary angiogram was normal. The operative procedure consisted of resection of a part of prolapsing A2 and annuloplasty using a Carpentier-Edwards annular ring; the chordae were not re-implanted during this procedure. The patient remained asymptomatic postoperatively, and he presented to our outpatient clinic seven years later for follow-up on his cardiac condition.

**Figure 1:**
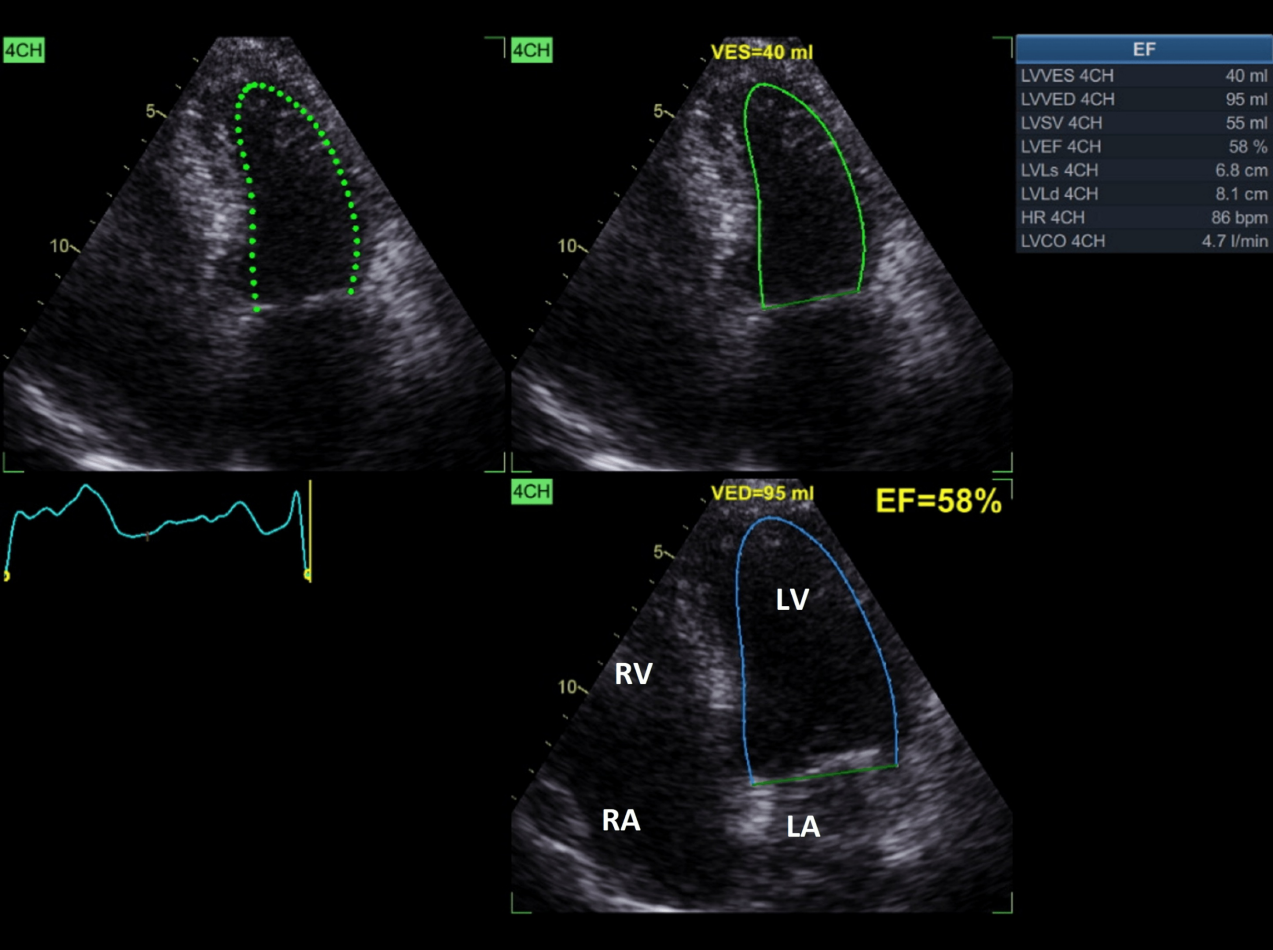
Apical four-chamber view showing automatic left ventricular ejection fraction at 58%. 4CH = four chambers; LA = left atrium; LV = left ventricle; RA = right atrium; RV = right ventricle; VED = volume end diastole; VES = volume end systole; EF = ejection fraction

On presentation to the ambulatory clinic, the patient was asymptomatic; his medications consisted of the antihypertensive drugs perindopril 10 mg plus indapamide 1.5 mg and aspirin taken orally on a daily basis. On physical examination, the heart rate was 75 beats per minute and regular, blood pressure was 125/75 mmHg, respiratory rate was 16 breaths per minute, and he was afebrile. There was no neck vein distension. On auscultation, a systolic murmur grade II/VI was heard over the mitral area, and the lungs were clear. Abdominal examination was unremarkable. The extremities showed adequate pulses with no leg edema. Due to the findings of the physical examination and the late follow-up of the patient, a transthoracic echocardiogram with color Doppler was requested. The echocardiogram showed a mildly dilated left atrium with an anteroposterior diameter of 4.5 cm, mild concentric hypertrophy of the LV with a left ventricular internal diameter in systole at 3.6 cm, and a relative wall thickness (RWT) of 0.46. Additionally, the LVEF was normal at 58% (Figure 1).

**Figure 2:**
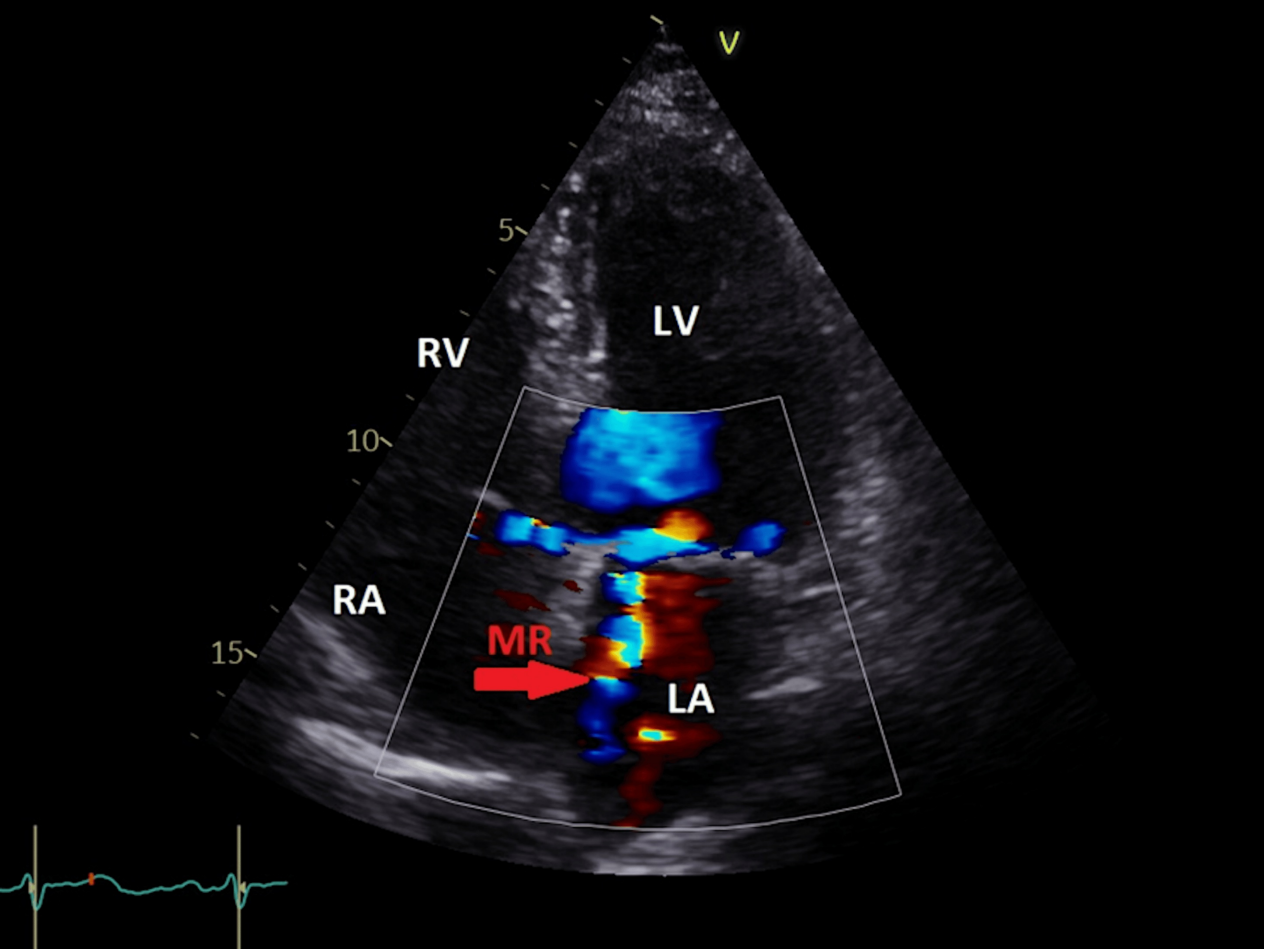
Apical four-chamber view showing grade II mitral regurgitation by color flow (red arrow). LA = left atrium; LV = left ventricle; RA = right atrium; RV = right ventricle

The aortic valve was normal. The mitral valve showed that both leaflets were pliable. There was a mitral annular ring in place, and grade II mitral regurgitation was observed (Figure 2).

**Figure 3:**
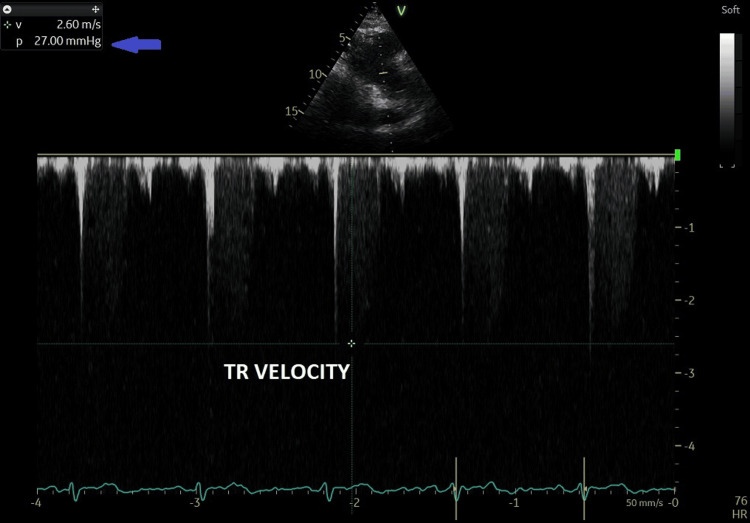
Tricuspid regurgitation velocity recording using continuous wave Doppler at 27 mmHg (blue arrow). TR VELOCITY = tricuspid regurgitation velocity; p = pressure gradient; V = velocity

The RA and the right ventricle (RV) were normal in size; the right ventricular systolic function was normal with a tricuspid annular excursion (TAPSE) at 17 mm. The pulmonary valve was normal. The tricuspid valve showed grade I regurgitation with a measured tricuspid regurgitation velocity of 2.7 m/sec (Figure 3), allowing the calculation of systolic pulmonary artery pressure (SPAP) at 34 mmHg. The right atrial pressure was estimated at 5 mmHg, as the inferior vena cava was normal in size at 1.6 cm and collapsed >80% with inspiration.

**Figure 4:**
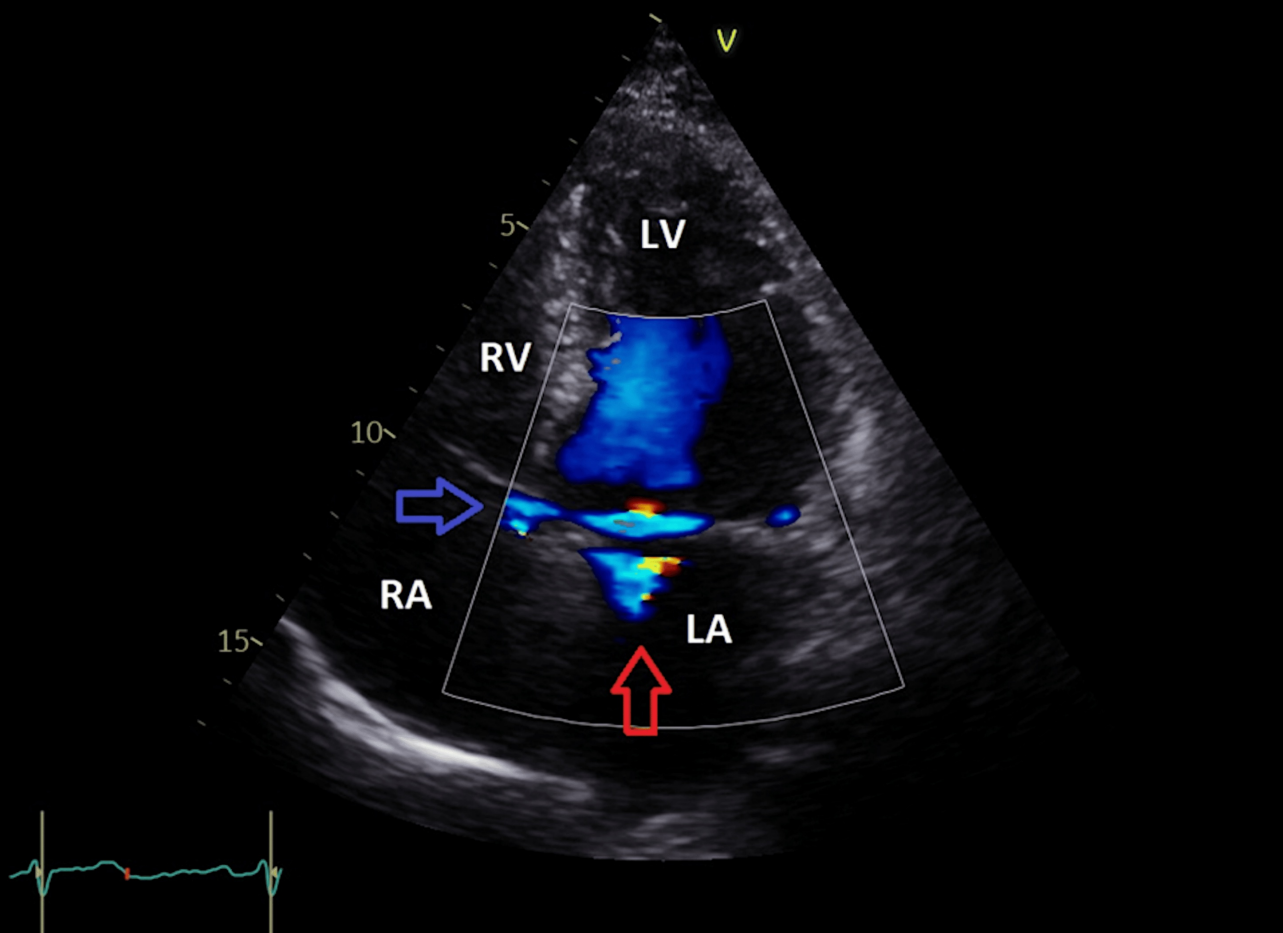
Apical four-chamber view showing mitral regurgitation jet (red arrow) with color aliasing in the right atrium (blue arrow). LA = left atrium; LV = left ventricle; RA = right atrium; RV = right ventricle

In the apical four-chamber view, there was color Doppler aliasing noted in the RA just above the septal tricuspid leaflet, and it was not related to the tricuspid regurgitation (Figure 4, Video 1). This color aliasing in the RA indicates the exit of the Gerbode defect (a shunt into the RA).

**Video 1:** Apical four-chamber view showing grade II mitral regurgitation by color flow and color flow aliasing in the right atrium resulting from a left ventricular to right atrium shunt (acquired Gerbode defect); note that color flow aliasing clearly shows the shunt from the left ventricle to the right atrium.

**Figure 5:**
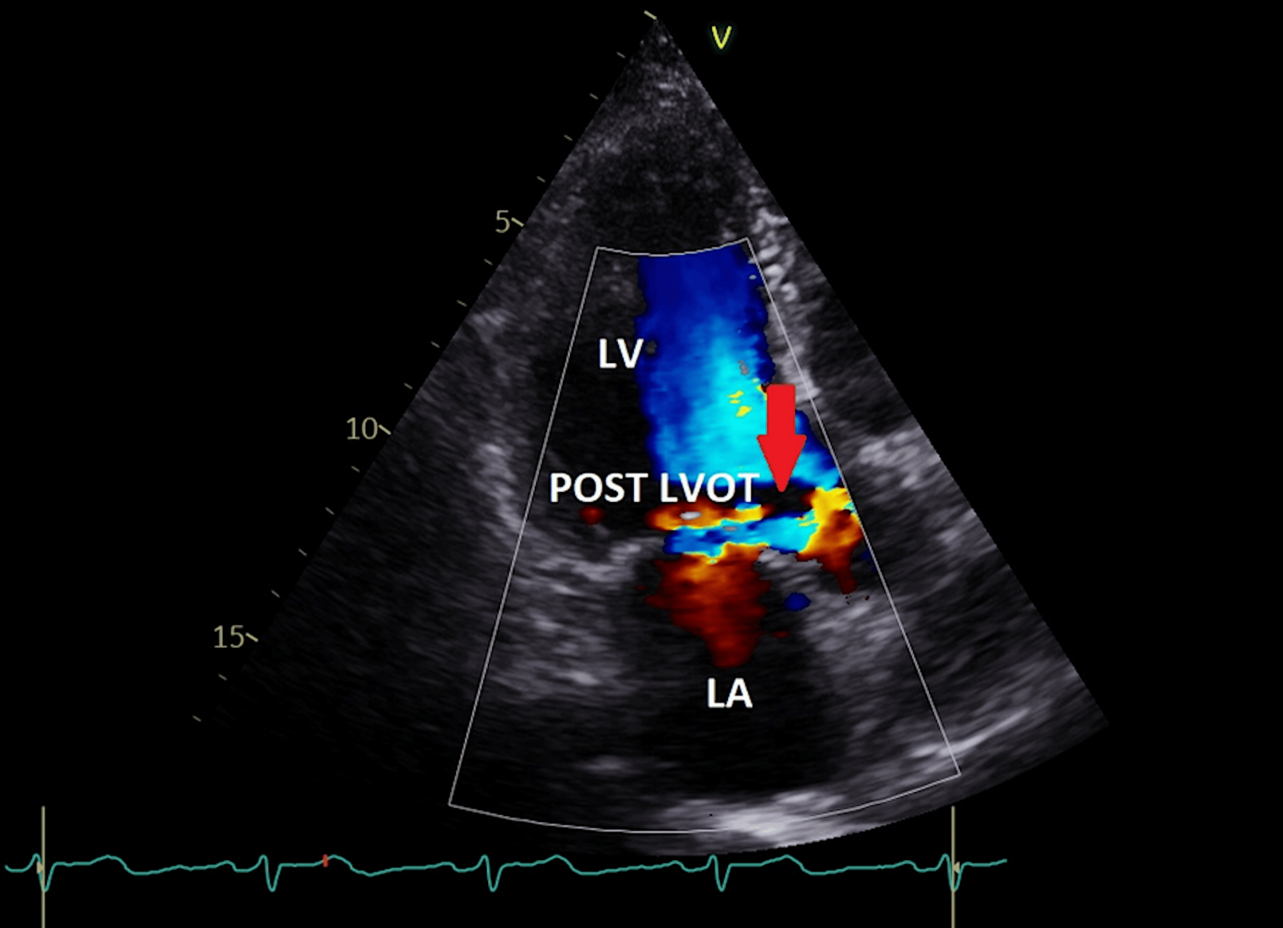
Apical three-chamber view showing color flow aliasing in the posterior part of the LVOT (red arrow) with normal color flow in the anterior part of the LVOT. This color aliasing in the LVOT marks the onset of the Gerbode defect from the left ventricle to the right atrium. LA = left atrium; LV = left ventricle; POST LVOT = posterior left ventricular outflow tract

In the apical long-axis view, color Doppler aliasing was also noted in the posterior part of the left ventricular outflow tract (LVOT) with normal color flow mapping in the anterior LVOT. This raised suspicion of an LV-RA shunt originating at the LVOT (Figure 5, Video 2).

**Video 2:** Apical three-chamber view showing color flow aliasing in the posterior part of the left ventricular outflow tract (LVOT) with normal color flow in the anterior part of the LVOT. This color aliasing in the LVOT marks the onset of the Gerbode defect from the left ventricle to the right atrium.

**Figure 6:**
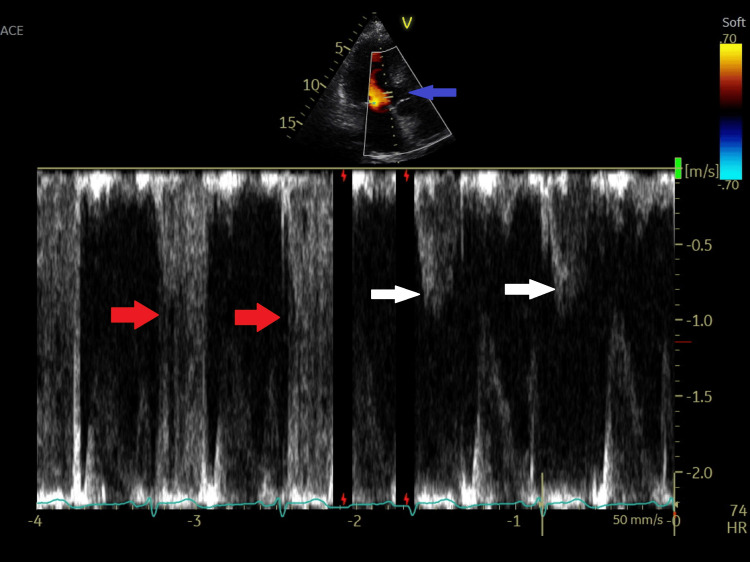
Pulsed Doppler wave recording from the posterior left ventricular outflow tract (red arrow) to the anterior LVOT ventricular outflow tract showing aliasing velocity in the posterior LVOT and normal velocity at the anterior LVOT (white arrow). The blue arrow indicates the final placement of the pulsed sample volume at the anterior LVOT.

Scanning the LVOT with pulsed wave (PW) Doppler in the apical long-axis three-chamber view from its posterior to anterior part showed aliasing at its posterior part below the posterior aortic cusp (non-coronary cusp) and normal flow below the anterior cusp (right coronary cusp) (Figure 6).

No VSD was noted by color flow (Video 1). Due to the suspicion of an acquired Gerbode defect, further scanning using color Doppler mapping was done from the apical four-chamber view, showing an LV-RA shunt originating from the LVOT (Video 3). Color Doppler aliasing confirmed communication between the LV and RA just above the tricuspid septal leaflet (Video 1). The Doppler aliasing (by color and PW) below the posterior aortic cusp (Figures 5, 6) represents the entry (or the start) of the Gerbode defect (channel), and the exit is in the RA just above the septal tricuspid leaflet (Figure 4, Video 1), as documented by color flow aliasing in the right atrium just above the tricuspid septal leaflet.

**Video 3:** On further scanning using color Doppler mapping from the apical four-chamber view, an LV-RA shunt originating from the left ventricular outflow tract was noted.

In our patient, the septal tricuspid leaflet was not affected, indicating a supravalvar Gerbode defect type 1 (or a direct Gerbode defect). The LV-RV shunt was not significant by color flow mapping; this was also documented by a normal calculated (pulmonary output/systemic output) QP/QS at 0.9 (LVOT velocity time integral (LVOT VTI) = 23 cm, LVOT diameter = 2.0 cm, right ventricular outflow tract (RVOT) VTI = 22.6 cm, RVOT diameter = 1.9 cm). The QP/QS was calculated using the following continuity equation formula: \(QP= π*(RVOT-diameter)^2^*(RVOT VTI)/4 and QS= π*(LVOT diameter)^2^*(LVOT VTI)/4\).

Additionally, there was no dilatation of the RA and RV. Pulmonary artery pressure was calculated at 34 mmHg. The patient was advised to undergo a transesophageal echocardiogram (TEE) for better visualization of the defect, but he refused and opted for a less invasive test, arguing that the TEE he had done before his cardiac surgery seven years ago was a traumatizing experience. Consequently, a cardiac MRI was requested and showed an LV-RA shunt just above the tricuspid septal leaflet. The LV-RA shunt was labeled as minimal with no hemodynamic consequences (Video 4).

**Video 4:** Cardiac MRI showing a minimal shunt from the left ventricle to the right atrium just above the tricuspid valve. Cardiac MRI = cardiac magnetic resonance imaging

The patient was advised to follow up every six months with an echocardiogram and color Doppler to assess progression. He was also instructed on the importance of reporting chest symptoms like chest pain, dyspnea while exercising, or palpitations.

## Discussion

Surgical mitral valve repair remains the main treatment for severe primary mitral regurgitation, especially in patients with prolapse and elongated, myxomatous valve leaflets [2-4]. Usually, the surgical repair of the posterior leaflet is more technically feasible and durable, and this is why the recommendation of the American College of Cardiology (ACC)/American Heart Association (AHA) in the case of only posterior leaflet prolapse is to proceed with surgical mitral valve repair and not valve replacement [1-4].

**Figure 7:**
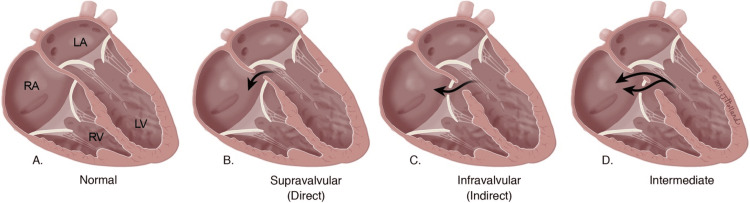
Comparison of normal heart to hearts with Gerbode defect. (A) Normal heart. (B) Supravalvular defect involving the membranous portion of the septal wall, superior to the septal leaflet of the tricuspid valve. (C) Infravalvular defect involving the membranous portion of the septal wall, below the septal leaflet. (D) Both supravalvular and infravalvular defects with the septal leaflet of the tricuspid valve. LA = left atrium; LV = left ventricle; RA = right atrium; RV = right ventricle Illustration by Jessica Holland © 2016. Reproduced from Saker et al. [9] under the CC-BY-NC-ND 4.0 license

Repair of the anterior mitral leaflet is more technically demanding and has a higher failure rate over time; in fact, in one cohort, only 55.6% of anteriorly prolapsed valves were amenable to repair, and 44.4% received replacement [5,6]. The mortality rate of surgical valve repair in carefully selected patients should not exceed 1%; the morbidity can reach up to 8%, and all the complications are the same as those seen with cardiac surgery when patients are put on a bypass machine [8]. In one study, the recurrence rate of moderate mitral regurgitation (grade > 2/4) was 3.6 per year [7]. One rare complication of the surgical mitral valve repair is the acquired Gerbode defect [11]. Figure 7 illustrates the types of Gerbode defects and their relation to the tricuspid valve, reproduced from Saker et al. [9].

Since it is a rare complication, it should be ruled out during echocardiography in patients post-mitral and aortic valve procedures, looking for abnormal color aliasing in the RA and LVOT [12]. Other imaging techniques, such as TEE, cardiac MRI, and cardiac CT, will help confirm the diagnosis [11,12]. Left ventricular angiography during a diagnostic coronary angiogram will also confirm the diagnosis, but this technique has long been replaced with the non-invasive imaging modalities (echocardiogram, cardiac MRI, and cardiac CT) [11,12]. When patients with severe primary mitral regurgitation cannot tolerate a surgical procedure, then percutaneous mitral valve repair becomes indicated [13].

In our patient, color Doppler aliasing in the RA, unrelated to tricuspid regurgitation, raised suspicion of this defect. Furthermore, color Doppler aliasing in just one part of the LVOT (the posterior part in our case) raised suspicion of this defect; the LVOT was the entry site of the communication (shunt), and the exit site was the RA, just above the tricuspid septal leaflet. Our patient had a non-significant LV-RA shunt hemodynamically, as evidenced by a normal QP/QS of 0.9 and normal right atrial and right ventricular size; the SPAP was calculated at 34 mmHg. Furthermore, our patient had failure of surgical mitral valve anterior leaflet prolapse repair, which is one of the long-term complications of the repair of the anterior mitral leaflet [5-7]. Since our patient was asymptomatic, he was advised to follow up every six months with serial echocardiograms, and he was instructed on the importance of reporting new-onset symptoms like chest pain, dyspnea on exercise, or palpitations. If there is an increase in the severity of mitral regurgitation, an increase in cardiac chamber size, a decrease in left ventricular (LV) function, or the development of new cardiac symptoms, then the decision regarding mitral valve re-intervention and closure of the Gerbode defect will be made collaboratively with the cardiac team and after obtaining patient consent.

## Conclusions

Surgical mitral valve repair for severe primary regurgitation due to prolapse with myxomatous leaflet degeneration is recommended in patients with no surgical contraindications. Percutaneous mitral valve repair is recommended for symptomatic patients with severe primary mitral regurgitation who are at high surgical risk. Repair of the posterior mitral leaflet prolapse is more effective and durable than the repair of the anterior leaflet. The complication rate of surgical mitral repair is low. Post-surgical repair recurrence of mitral regurgitation warrants meticulous follow-up with serial echocardiograms to detect signs indicating the need for re-intervention. Echocardiographers should also always be on the lookout for the very rare Gerbode defect post-surgical mitral valve repair; in case the Gerbode defect is hemodynamically significant, leading to cardiac symptoms, then percutaneous closure is advised.
